# *Inonotus obliquus* fermentation product improves growth performance and meat quality probably through intestine and antioxidant capacity enhanced by gut microbes and metabolites regulation in rabbits

**DOI:** 10.1186/s42523-025-00427-7

**Published:** 2025-06-09

**Authors:** Lin Zhang, Zhiguo Fei, Yaling Ding, Yajia Zhang, Zhiyong Ding, Yueyan Huang, Junkun Wang, Gongyan Liu, Liya Bai, Jiaqiang Wu

**Affiliations:** 1 Institute of Animal Science and Veterinary Medicine, Shandong Academy of Agricultural Sciences, Jinan, Shandong China; 2 Key Laboratory of Livestock and Poultry Multi-Omics of MARA, Jinan, Shandong China; 3 College of Life Science and Food Engineering, Hebei University of Engineering, Handan, Hebei China; 4 Qilu Animal Health Products Co., Ltd., Jinan, Shandong China; 5 College of Animal Science and Technology, Hebei Normal University of Science and Technology, Qinhuangdao, Hebei China; 6 Qinhuangdao Gaotong Biotech Co., Ltd., Changli, Hebei China

**Keywords:** *Inonotus obliquus* fermentation product, Rabbit, Intestinal microbiome, Metabolome, Antibiotic alternative

## Abstract

**Background:**

*Inonotus obliquus* is a medicinal edible fungus that contains a variety of biologically active ingredients and has multiple physiological effects. When supplemented in avian diet, *Inonotus obliquus* has proved to be beneficial. However, information regarding these effects on mammals is scanty. The present study aims to investigate the effect of supplementation of *Inonotus obliquus* fermentation product (IOFP) on the growth performance, antioxidant capacity, meat quality, intestinal function and gut microbiota of rabbit exploratorily, which may act as an important feed additive and also as an antibiotic alternative with its medicinal properties.

**Results:**

Dietary supplementation of IOFP increased body weight (*P* < 0.01) at the initial 21 d and improved feed efficiency throughout the 35 d experimental period when compared to control group. At the same time it was observed that meat quality and carcass parameters improved upon supplementation of IOFP. Additionally, IOFP supplementation resulted in significant increases (*P* < 0.05) in total antioxidant capacity (T-AOC), superoxide dismutase (SOD), and nitric oxide (NO) activity or concentration in the serum and muscle. The crypt depth decreased significantly, whereas the villus height/crypt depth (V/C) value increased (*P* < 0.05). The concentration of secrete IgA (sIgA) of the intestine also increased (*P* < 0.05). IOFP supplementation significantly increased the fold change expression of *Claudin 1*, *Occludin*, *ZO1*, and *ZO2* (*P* < 0.05) when compared to the respective gene expression levels of the duodenum and jejunum tissues of control group. Further study on cecum microbiota revealed that IOFP supplementation increased the microbiota diversity by increasing the number of beneficial bacteria and reducing the numbers of pathological bacteria. It was observed that cecum metabolites produced in the treated group were related to antioxidants, antiinflammation and antidepressive effects. The harmful metabolites related to fat deposition, loss of appetite and cytotoxic conditions decreased. Pearson’s correlation studies between different bacteria and metabolites revealed that the metabolites produced were regulated by the beneficial and harmful bacteria respectively.

**Conclusions:**

IOFP enhanced intestinal morphology and function, and organismic antioxidant capacity, probably by increasing the concentration of beneficial microbiota and metabolites resulting in improvement of body weight, feed efficiency, and parameters related to meat quality and carcass traits of rabbits.

**Graphical Abstract:**

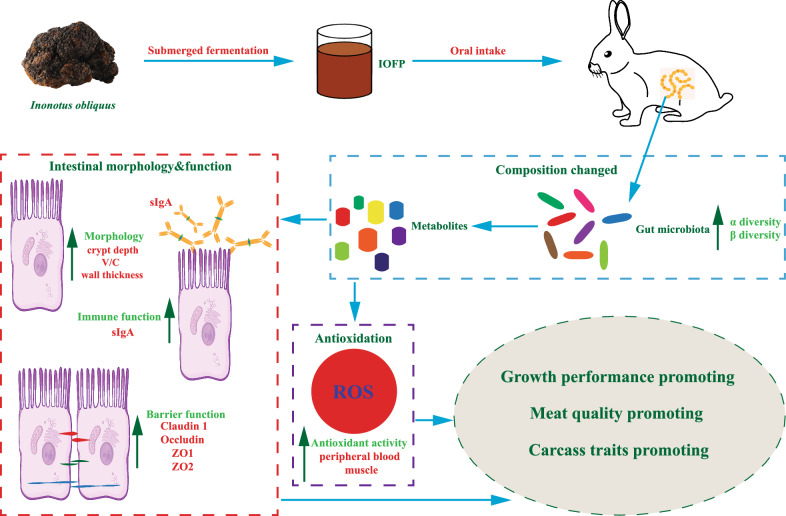

**Supplementary Information:**

The online version contains supplementary material available at 10.1186/s42523-025-00427-7.

## Background

Rabbit meat has a distinctive flavor and offers high nutritional value, making it a sought-after choice, especially in regions such as China and South Korea. Its popularity continues to surge each year, driven by its culinary versatility and health benefits [1, 2]. An increase in demand means not just heightened production but also an enhancement in meat quality [3]. Intensive farming methods are pivotal for achieving high production rates. However, an excessive focus on maximizing density and growth rates has resulted in a decline in meat quality (such as lower early postmortem pH value, increased drip loss, and higher shear force). This approach has also increased the risk of diseases (such as elevated mortality rates during the weaning period, intensified respiratory disease, and enteritis). Following the ban on antibiotics in feed in China, a further decline in disease resistance and growth performance of animals occurred [4, 5]. Thus, achieving both quantity and quality improvements of rabbit meat under antibiotic-free conditions is crucial. This not only enhances the commercial and industrial economic benefits of the products, but also serves as the key to overcome the bottleneck in the breeding industry.

The prolonged use of antibiotics in animal production yields promising industrial economic benefits due to their antimicrobial and growth-promoting effects. However, ongoing challenges persist, such as bacterial resistance, antibiotic residues, and environmental contamination, which demand immediate attention. Extensive research has been conducted on safe, green, and environmentally friendly alternatives [6, 7]. Currently, an increasing number of natural materials or products are being utilized in animal production, which has demonstrated efficacy in enhancing growth performance, reducing disease incidence, and minimizing antibiotic usage [8]. *Inonotus obliquus* has been used in the treatment of hypertension, gastric cancer, and various chronic and malignant diseases [9]. Modern medical research confirms that *Inonotus obliquus* exhibits immunomodulatory, antitumor, antioxidant, antidiabetic, antiviral, and antibacterial activities [10], establishing it as an outstanding fungus with medicinal benefits. However, its application in animal husbandry remains in infancy. In 2017, *Inonotus obliquus* fermentation product (IOFP)-derived from submerged mycelium culture-was initially added to chicken basal diet. The results showed significant improvements in the growth and immune performance of chickens [11]. When IOFP was used simultaneously with a vaccine as an immunopotentiator, it resulted in stronger and earlier humoral immune responses, as evidenced by increased total and neutralizing antibody titers [12]. Moreover, IOFP exhibits immunomodulatory activity in chickens, facilitating the transformation of immune responses towards a more profitable type [12]. Subsequently, IOFP were added in duck diets, leading to significant enhancements in growth performance, immune function, and antioxidant capacity among birds [13]. These results confirm the efficacy of IOFP as a highly effective feed additive and immunopotentiator for poultry; however, information regarding its effects on mammals remains limited. Recently, the effect of IOFP on immune performance in Hyla rabbits was examined, revealing significant enhancements in the development of immune organs and specific immune responses [2, 9]. Nonetheless, the underlying mechanism remains unclear. Moreover, the effects of IOFP on growth performance, antioxidant activity, meat quality, intestinal function, and gut microbiota of rabbits remain unknown. In this study, Hyla rabbits were fed a diet enriched with IOFP. Various aspects, including growth performance, antioxidant capacity, meat quality, intestinal function, and gut microbiota, were assessed to comprehensively evaluate the effects and elucidate the mechanism of IOFP in rabbits. This study could contribute to the development of innovative and environmentally friendly feed additives and immunopotentiators suitable for mammals.

## Results

### Chemical composition of IOFP

Through ultra high performance liquid chromatography-tandem mass spectrometry (UHPLC-MS/MS) analysis, a total of 916 constituents were found in the IOFP test solution. Based on database search and comparison, 208 bioactive constituents were characterized, mainly including amino acid, nucleotide, fatty acid, vitamin, polysaccharides, carboxylic acid, polyphenols and other components (details were listed in Supplementary Information Table S1).

### Effect of IOFP on rabbit productivity

Table 1 summarizes the effects of IOFP on the growth performance of rabbits. All rabbits in this study initially had similar BW (Table 1, *P* > 0.05). The average daily gain (ADG) in the IOFP group exhibited a significant increase (*P* < 0.01) during the initial 21 d of experimental period. However, no significant difference (*P* > 0.05) was observed during the subsequent 14 d of experimental period, when compared to the control group. In contrast to the ADG, average daily feed intake (ADFI) and feed-to-gain ratio (F/G) decreased significantly (*P* < 0.05) in IOFP group when compared to the control group during 35 d experimental period. These findings suggest that dietary supplementation with IOFP enhanced body weight (BW) gain during the initial phase and feed efficiency (ADG/ADFI) during the entire trial period.

**Table 1:** Effect of *Inonotus obliquus* fermentation product (IOFP) on rabbit productivity

Item	Control^1^	IOFP^2^	SEM	*P* value
Initial BW, g	869.28	873.25	20.23	0.451
Final BW, g	2216.46^a^	2328.71^b^	53.76	0.008
d 1–7				
ADG, g	33.68^a^	40.41^b^	0.35	0.002
ADFI, g	111.52^a^	103.99^b^	25.03	0.001
F/G	3.31^a^	2.57^b^	0.19	< 0.001
d 8–14				
ADG, g	45.15^a^	48.26^b^	0.29	0.002
ADFI, g	219.83^a^	207.30^b^	29.18	0.001
F/G	4.87^a^	4.30^b^	0.27	0.006
d 15–21				
ADG, g	33.29^a^	39.62^b^	0.30	0.002
ADFI, g	168.33^a^	171.51^b^	23.45	0.010
F/G	5.06^a^	4.33^b^	0.24	0.009
d 22–28				
ADG, g	33.69	33.90	0.24	0.482
ADFI, g	174.77^a^	167.52^b^	22.18	0.025
F/G	5.19^a^	4.94^b^	0.29	0.028
d 29–35				
ADG, g	34.63	34.19	0.19	0.055
ADFI, g	217.75^a^	183.30^b^	25.13	0.020
F/G	6.29^a^	5.36^b^	0.32	0.023

*BW* body weight; *ADG* average daily gain; *ADFI* average daily feed intake; *F/G* feed to gain ratio; *SEM* standard error of means

^1^Control, basal diet

^2^IOFP, basal diet supplemented with IOFP (0.8% (w/w); Qinhuangdao Gaotong Biotech Co., Ltd, Changli, China)

^a,b^Different lower-case superscript letters mean significantly difference (*P* < 0.05). Mean values are based on surviving rabbits per replicate and 10 replicates per group

During the transition from breastfeeding to complete solid-state feeding, rabbits typically experience a high mortality rate. During the experimental period, the mortality rate in the control group was 15%, whereas for IOFP group was only 2.5%, which indicated significant decrease in mortality rate (*P* < 0.05) (Table 2).

**Table 2:** Effect of *Inonotus obliquus* fermentation product (IOFP) on rabbit mortality

Item	Control^1^	IOFP^2^	*P* value
Total	40	40	–
Death	6^3^	1^4^	–
Mortality	15%^a^	2.5%^b^	0.048

^1^Control, basal diet

^2^IOFP, basal diet supplemented with IOFP (0.8% (w/w); Qinhuangdao Gaotong Biotech Co., Ltd, Changli, China)

^3^6 dead rabbits in control group died at three experimental periods: 2 at 1–7 d, 2 at 15–21 d and 1 at 29–35 d of experimental period

^4^1 dead rabbit in IOFP group died at 8–14 d of experimental period

^a,b^Different lower-case superscript letters mean significantly difference (*P* < 0.05). Mean values are based on surviving rabbits per replicate and 10 replicates per group

### Effect of IOFP on carcass traits and meat quality of rabbits

After 35 d fed with basal diet supplemented with IOFP, higher pre-slaughter weight, commercial slaughter ratio, semi and full clean slaughter ratio of treated rabbits were significantly higher (*P* < 0.05) when compared to the ones in control group (Table 3). These findings indicate that IOFP enhanced the commercial value of rabbits.

**Table 3:** Effect of *Inonotus obliquus* fermentation product (IOFP) on rabbit carcass traits

Item	Control^1^	IOFP^2^	SEM	*P* value
Pre-slaughter weight, g	2128.30^a^	2225.00^b^	70.21	0.007
Commercial carcass weight, g	1225.26^a^	1314.98^b^	45.60	0.014
Commercial slaughter ratio, %	57.57^a^	59.10^b^	0.42	0.039
Semi clean carcase weight, g	1093.52^a^	1196.83^b^	39.47	0.007
Semi clean slaughter ratio, %	51.38^a^	53.79^b^	0.37	0.037
Full clean carcase weight, g	986.25^a^	1069.78^b^	37.23	0.001
Full clean slaughter ratio, %	46.34^a^	48.08^b^	0.28	0.013

*SEM* standard error of means

^1^Control, basal diet

^2^IOFP, basal diet supplemented with IOFP (0.8% (w/w); Qinhuangdao Gaotong Biotech Co., Ltd, Changli, China)

^a,b^Different lower-case superscript letters mean significantly difference (*P* < 0.05)

Mean values are based on surviving rabbits per replicate and 10 replicates per group

Table 4 summarizes the effects of IOFP supplementation on rabbit meat quality. IOFP dietary supplementation did not cause significant difference on the the pH, brightness, and yellowness (*P* > 0.05) of longissimus thoracis et lumborum (LTL) muscle. However, supplementation significantly affected redness, cooking loss ratio, drop loss, water loss, and shear force parameters when compared to control group (*P* < 0.05). The above results indicated that addition of IOFP to the diet increased the meat quality parameters significantly.

**Table 4:** Effect of *Inonotus obliquus* fermentation product (IOFP) on rabbit meat quality

Item	Control^1^	IOFP^2^	SEM	*P* value
pH	5.76	5.72	0.02	0.804
Brightness (L*)	51.27	52.20	0.37	0.069
Redness (a*)	5.34^a^	6.50^b^	0.30	0.032
Yellowness (b*)	7.45	7.34	0.15	0.414
Drop loss, %	1.92^a^	1.60^b^	0.10	0.048
Water loss, %	6.89^a^	6.03^b^	0.20	0.026
Cooking loss ratio, %	29.34^a^	31.79^b^	0.53	0.020
Shear force, kgf	3.11^a^	2.96^b^	0.01	0.003

*SEM*standard error of means

^1^Control, basal diet

^2^IOFP, basal diet supplemented with IOFP (0.8% (w/w); Qinhuangdao Gaotong Biotech Co., Ltd, Changli, China)

^a,b^Different lower-case superscript letters mean significantly difference (*P* < 0.05)

Mean values are based on surviving rabbits per replicate and 10 replicates per group

### Effect of IOFP on serum and muscle antioxidant capacity of rabbits

**Fig. 1:**
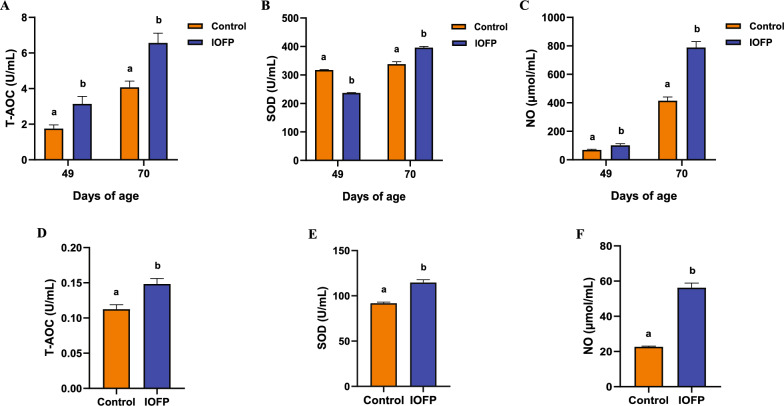
Effect of *Inonotus obliquus* fermentation product (IOFP) on serum and muscle antioxidant capacity of rabbits. The serum T-AOC (**A**), SOD (**B**) and NO (**C**) on 49 and 70 d of age, and muscle T-AOC (**D**), SOD (**E**) and NO (**F**) on 70 d of age are displayed. Different lowercase letters mean significantly difference (*P* < 0.05) between IOFP and control group. The error bars are based on the standard error of means

Figure 1A–C shows the effects of IOFP on serum antioxidant indices. Total antioxidant capacity (T-AOC) activity of the IOFP group significantly increased when compared to the control group at 49 and 70 d of age (*P* < 0.05). In contrast to the trend observed in T-AOC, the superoxide dismutase (SOD) value in IOFP-fed rabbits was less at 49 d of age and greater at 70 d of age when compared to respective serum mean values of the control group (*P* < 0.05). However, when the serum SOD and T-AOC values were compared between the 49 and 70 d of age of the control group, the difference was not significant (*P* > 0.05). The trend in and nitric oxide (NO) concentration was combine with T-AOC result, being higher in the IOFP group than in the control group at both time points (*P* < 0.05). The same parameters were evaluated in muscle tissues collected at 70 d of age, it was observed that the T-AOC, SOD and NO activity or concentrations increased in muscles of IOFP group compared to control group. Figure 1D–F depicts the activities of T-AOC, SOD, and NO in the IOFP group were significantly higher than those in the control group (*P* < 0.05).

### Effect of IOFP on intestinal tissue morphology and secrete IgA (sIgA) of rabbits

The addition of IOFP to the basal diet led to varying degrees of improvement in intestinal villus height, crypt depth, and V/C (Table 5). In IOFP-treated rabbits, the crypt depth decreased by 29.9% (*P* < 0.05), while the V/C value increased by 31.33% (*P* < 0.05), compared to untreated animals. These findings suggest that IOFP significantly improved the intestinal morphology.

**Table 5:** Effect of *Inonotus obliquus* fermentation product (IOFP) on intestinal tissue morphology

Item	Control^1^	IOFP^2^	SEM	*P* value
Villi height, μm	528.97	539.05	6.74	0.081
Crypt depth, μm	132.94^a^	93.17^b^	6.89	0.013
Wall thickness, μm	166.39^a^	104.42^b^	10.75	0.014
V/C	3.99^a^	5.81^b^	0.325	0.011

*V/C* villi height to crypt depth; *SEM* standard error of means

^1^Control, basal diet

^2^IOFP, basal diet supplemented with IOFP (0.8% (w/w); Qinhuangdao Gaotong Biotech Co., Ltd, Changli, China)

^a,b^Different lower-case superscript letters mean significantly difference (*P* < 0.05)

Mean values are based on surviving rabbits per replicate and 10 replicates per group

**Fig. 2:**
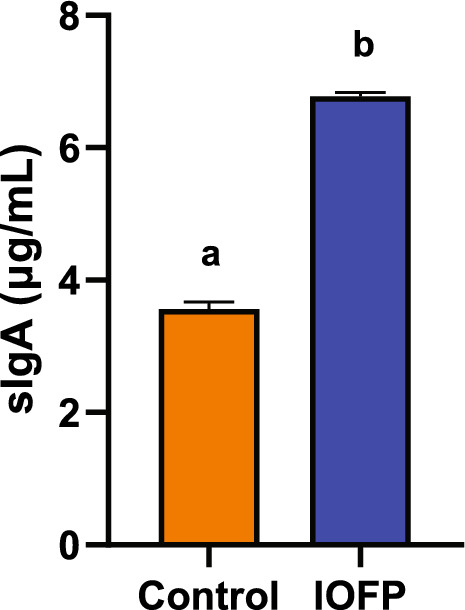
Effect of *Inonotus obliquus* fermentation product (IOFP) on intestinal secrete IgA (sIgA) of rabbits. The contents of intestinal sIgA on 70 d of age are displayed. Different lowercase letters mean significantly difference (*P* < 0.05) between IOFP and control group. The error bars are based on the standard error of means

Figure 2 depicts the intestinal sIgA content in rabbits. Dietary supplementation with IOFP significantly increased sIgA secretion than in the control group (*P* < 0.05), suggesting a beneficial effect of IOFP on intestinal mucosal immunity.

### Effect of IOFP on tight junction gene expression in rabbits

**Fig. 3:**
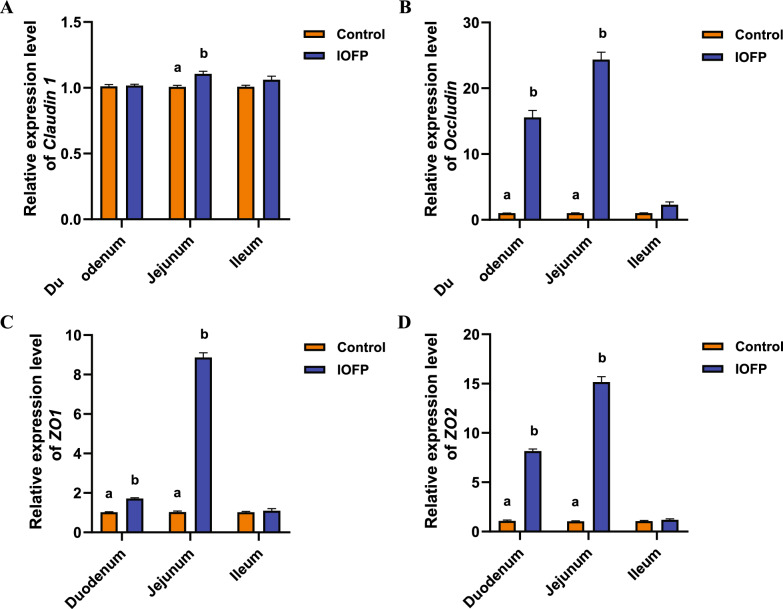
Effect of *Inonotus obliquus* fermentation product (IOFP) on expression of tight junction genes of rabbits. The relative fold change in the mRNA expression levels of *Claudin 1* (**A**), *Occludin* (**B**), *ZO1* (**C**) and *ZO2* (**D**) of duodenum, jejunum, ileum at 70 d of age in control and IOFP groups are displayed. Different lowercase letters mean significantly difference (*P* < 0.05) between IOFP and control group. The error bars are based on the standard error of means

The mRNA expression levels of intestinal barrier genes (*Claudin 1*, *Occludin*, *ZO1,* and *ZO2*) in the duodenum, jejunum, and ileum were measured separately in both groups. In the jejunum, the expression level of *Claudin 1* was significantly upregulated in IOFP group than that in the control group (*P* < 0.05), while in the duodenum and ileum, the expression levels were not significantly different when compared between the two groups (*P* > 0.05) (Fig. 3A). In contrast to *Claudin 1*, the expression levels of *Occludin*, *ZO1,* and *ZO2* were comparable influenced through the oral administration of IOFP (Fig. 3B–D). Specifically, the expression levels of *Occludin*, *ZO1*, and *ZO2* genes in the duodenum were upregulated by 14.83-fold (*P* < 0.05), 1.68-fold (*P* < 0.05), and 8.01-fold (*P* < 0.05), respectively than the expression levels of control group. In the jejunum, the upregulation of *Occludin*, *ZO1,* and *ZO2* expression levels was more obvious, reaching 23.58-fold (*P* < 0.05), 8.70-fold (*P* < 0.05), and 14.78-fold (*P* < 0.05), respectively. However, on comparing the fold change in the expression levels, there did not exhibit significant difference (*P* > 0.05) in the ileum.

### Effect of IOFP on cecal bacterial communities in rabbits

**Fig. 4:**
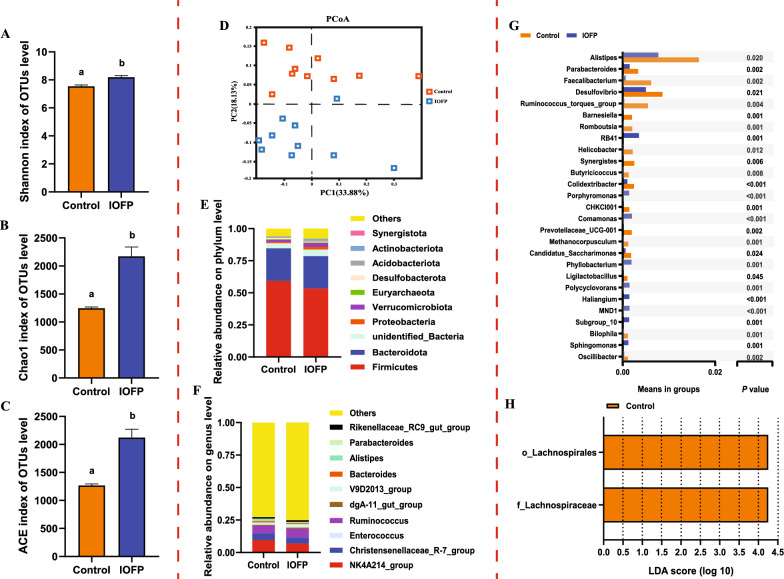
Effect of *Inonotus obliquus* fermentation product (IOFP) on cecal bacterial communities of rabbits. The alpha diversity is estimated by Shannon (**A**), Chao1 (**B**) and ACE (**C**). The beta diversity is estimated by principal co-ordinates analysis (PCoA) (**D**). The top 10 abundance of microbes in cecum on phylum (**E**) and genus (**F**) level are displayed. Significant different bacterial genera between control and IOFP group analyzed by T-test method is demonstrated in **G**. Biomarker microbes of control and IOFP group analyzed by linear discriminant analysis (LDA) effect size (LefSe) is displayed in **H** (LDA > 4). Different lowercase letters mean significantly difference (*P* < 0.05) between IOFP and control group. The error bars are based on the standard error of means

The α-diversity indices were significantly increased in the IOFP group (Fig. 4A-4C), including the diversity index (Shannon) (*P* < 0.05) and richness estimators (Chao1 (*P* < 0.05) and ACE (*P* < 0.05)) than those in the control group [14]. Figure 4D shows the principal coordinate analysis (PCoA) results, which highlight the distinct clustering of each group in different orientations. This suggests that dietary supplementation with IOFP not only enhanced the diversity of the gut microbiota but also induced alterations in microbial composition.

Microbial composition analysis (Fig. 4E, F) revealed the predominant bacteria at the phylum level in the cecum to be Firmicutes, Bacteroidota, and Verrucomicrobiota. A significant increase was observed in the populations of bacteria belonging to phylum of Verrucomicrobiota and Acidobacteria (*P* < 0.05), while Desulfobacterota and Synergistota were significantly reduced (*P* < 0.05) when compared to the control group. At the genus level, *NK4A214_group*, *Christensenellaceae_R-7_group*, *Ruminococcus*, *V9D2013_group,* and *Rikenellaceae* in the top 10 microbes were the predominant genera (> 1%) in the cecum. The relative abundance of *Alistipes* and *Parabacteroides* (*P* < 0.05) was significantly lower in the IOFP group than in the control group.

Subsequently, a t-test was employed to analyze the significance of the relative abundance between groups at the genus level (Fig. 4G). Compared to the control group, dietary IOFP supplementation led to a significant decrease in the relative abundance of *Alistipes*, *Desulfovibrio*, *Helicobacter*, *Candidatus_Saccharimonas*, and *Ligilactobacillus* (*P* < 0.05) in the cecum, while *parabacteroides*, *Faecalibacterium*, *Ruminococcus_torques_group*, *Barnesiella*, *Romboutsia*, *Synergistes*, *Butyricicoccus*, *Colidextribacter*, *CHKCI001*, *Prevotellaceae_UCG-001*, *Methanocorpusculum*, *Bilophila*, and *Oscillibacter* were significantly decreased (*P* < 0.01). Meanwhile, the relative abundances of *RB41*, *Porphyromonas*, *Comamonas*, *Phyllobacterium*, *Polycyclovorans*, *Haliangium*, *MND1*, *Subgroup_10,* and *Sphingomonas* significantly increased (*P* < 0.01) in the IOFP group.

Furthermore, a linear discriminant analysis effect size (LefSe) was conducted to identify significantly responsive microbes in the basal diet supplemented with dietary IOFP. The results showed that one family (Lachnospiraceae) and one order (Lachnospirales), which were biomarkers in the control group, were detected with a linear discriminant analysis (LDA) > 4 (Fig. 4H).

### Effect of IOFP on cecal metabolites of rabbits

**Fig. 5:**
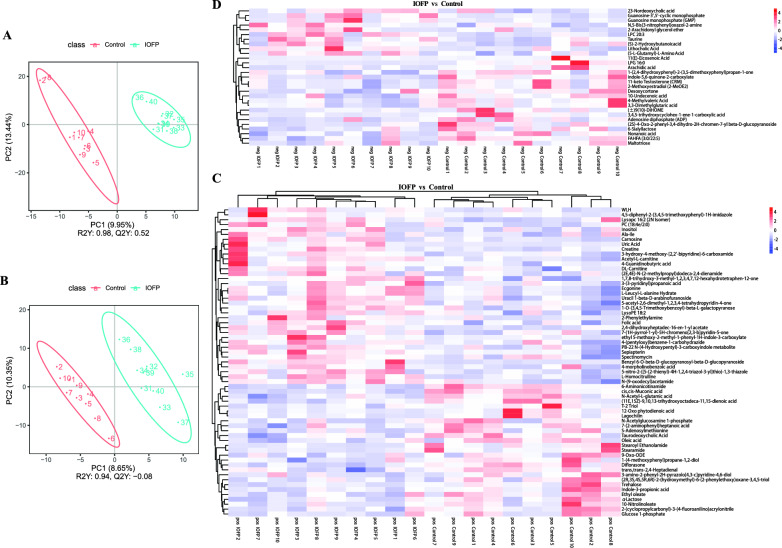
Effect of *Inonotus obliquus* fermentation product (IOFP) on cecal metabolites composition of rabbits. The composition of cecal metabolites under positive (**A**) and negative (**B**) ions of liquid chromatography-tandem mass spectrometry (LC–MS/MS) in control and IOFP group analyzed by partial least squares discriminant analysis (PLS-DA) is displayed. The differentially expressed metabolites under positive (**C**) and negative (**D**) ions of liquid chromatography-tandem mass spectrometry (LC–MS/MS) were identified between the IOFP and control groups, displayed in the form of heatmaps

Figure 5A, B presents the partial least squares discriminant analysis (PLS-DA), which revealed R2Y values of 0.98 and 0.94 for positive and negative ions, respectively. These values suggest that the model is stable and reliable. Moreover, the metabolic profiles of the specimens from the IOFP and control groups were clustered and distinguished. These findings suggested that supplementation of IOFP significantly modified the composition of cecal metabolite.

**Fig. 6:**
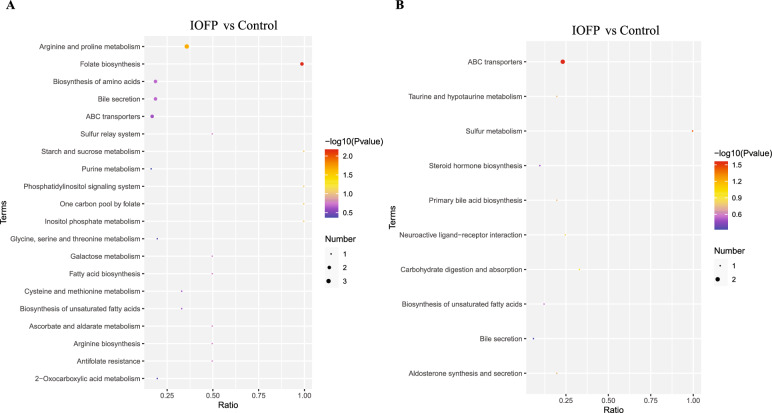
Kyoto Encyclopedia of Genes and Genomes (KEGG) analysis of differential metabolites. The enriched pathways under positive (**A**) and negative (**B**) ions are displayed. The significant enrichment pathway regarded as *P* < 0.05

**Fig. 7:**
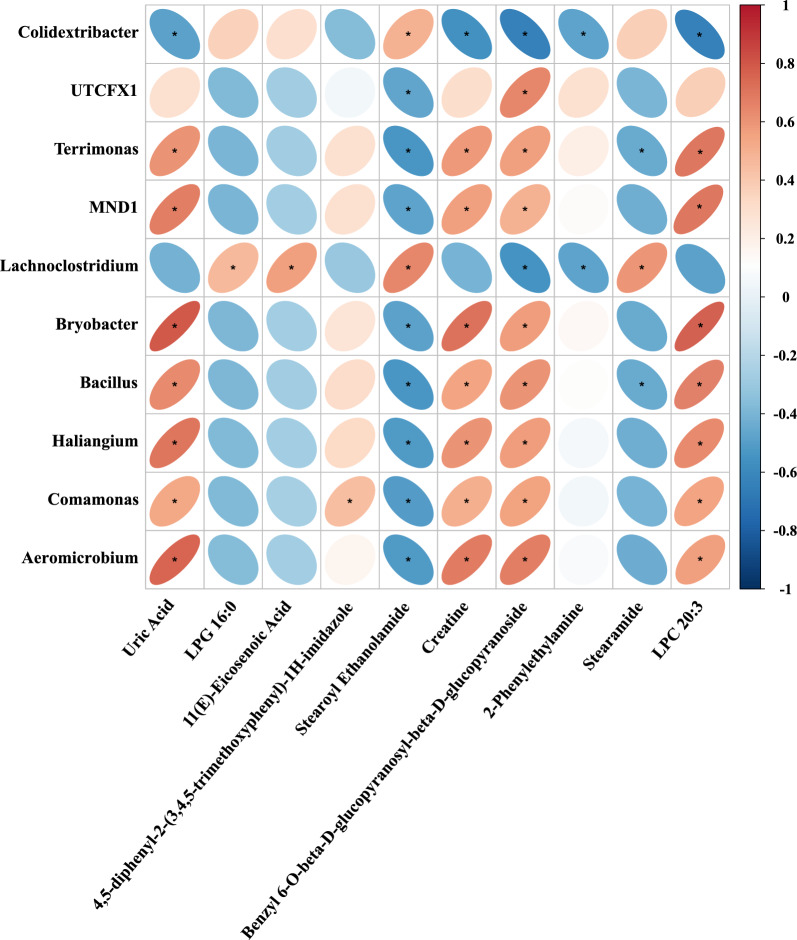
Correlation analysis of differential metabolites and microbes. The top 10 differential metabolites regarded as fold change (FC) and microbes regarded as *P* value at genus level are chosen to conducted correlation analysis with Pearson method. Red and blue ellipses mean positive and negative correlation, respectively. *means significantly correlations (*P* < 0.05) between metabolite and microbe

Overall, 92 metabolites differentially expressed were identified between the IOFP and control groups based on criteria-fold change (FC) > 1.2 and variable importance in the projection (VIP) > 1 or FC < 0.83 and *P* < 0.05 (Fig. 5C, D). Compared with the control group, the numbers of upregulated and downregulated metabolites were both 46 in the IOFP group. Table 6 lists the top 10 metabolites with the highest FC, including six metabolites with increased abundance (Uric Acid, 4,5-diphenyl-2-(3,4,5-trimethoxyphenyl)−1H-imidazole, Creatine, Benzyl 6-O-beta-D-glucopyranosyl-beta-D-glucopyranoside, 2-Phenylethylamine, Stearamide and LPC 20:3), and four metabolites with reduced abundance (LPG 16:0, 11(E)-eicosenoic acid, Stearoyl Ethanolamide and Stearamide). Subsequently, analysis of Kyoto Encyclopedia of Genes and Genomes (KEGG) indicated that differentially expressed metabolites mainly involved in four pathways (folate biosynthesis, arginine and proline metabolism, ABC transporters and sulfur metabolism), which was shown in Fig. 6. Figure 7 presents the results of correlation analysis between the top 10 differential metabolites and microbes at the genus level using the Pearson method. The correlation analysis revealed two distinct sections based on the correlation ratio: metabolites exhibiting increased relative abundance in the IOFP-supplemented group was positively correlated with numbers of *Aeromicrobium*, *Bacillus*, *Bryobacter*, *Comamonas*, *Haliangium*, *MND1*, *Terrimonas,* and *UTCFX1* populations*.* These metabolites displayed negative associations with *Colidextribacter* and *Lachnoclostridium*. In contrast, reduced metabolites in the IOFP-supplemented group showed a positive correlation with *Colidextribacter* and *Lachnoclostridium,* while exhibiting negative associations with the other eight microbes. Further analysis was conducted to determine *P* value. As shown in Fig. 7, multiple microbes influence changes in the relative abundance of metabolites.

**Table 6:** Differential metabolites in partial least squares discriminant analysis (PLS-DA)

NO	Metabolite^1^	Mode^2^	FC	*P* value
1	Uric Acid	+	5.35	0.001
2	LPG 16:0	−	0.29	0.004
3	11(E)-Eicosenoic Acid	−	0.31	0.026
4	4,5-diphenyl-2-(3,4,5-trimethoxyphenyl) −1H-imidazole	+	2.93	0.028
5	Stearoyl Ethanolamide	+	0.40	0.003
6	Creatine	+	2.37	0.010
7	Benzyl 6-O-beta-D-glucopyranosyl- beta-D-glucopyranoside	+	2.36	< 0.001
8	2-Phenylethylamine	+	2.31	0.019
9	Stearamide	+	0.46	0.015
10	LPC 20:3	−	2.12	0.015

*NO* number; *FC* fold change

^1^Metabolite, top 10 metabolites with highest fold change between treated and untreated groups

^2^Mode, positive (+) and negative (−) ions of LC–MS/MS in PLS-DA

Mean values are based on 1 rabbits per replicate and 10 replicates per group

## Discussion

*Inonotus obliquus* thrives in cold regions at high latitudes (45–50°). In response to cold climates, temperature fluctuations, and other environmental factors, multiple defense mechanisms evolved in *Inonotus obliquus*. The production of diverse bioactive substances, such as polysaccharides, polyphenols, inositols, sterols, and triterpenoids, serves for both defense and various physiological functions [15]. In previous studies, *Inonotus obliquus* demonstrated potential as an antibiotic substitute [13, 16–18]. As utilization of antibiotic alternatives hold significant practical value, employing the whole extracts to evaluate organism enhancement is more suitable, which is close to traditional folk medicine practices or the original form. Moreover, microbial fermentation can yield products that enhance the nutritional value of organisms by increasing nutrient bioavailability and decreasing the content of antinutritional factors [14]. Thus, in the present study, whole IOFP extracts were added to the basal diets of Hyla rabbits to assess their influence on growth performance, antioxidant activity, meat quality, intestinal function, and gut microbiota exploratorily. Incorporating IOFP supplemented the diet significantly enhanced body weight, antioxidant capacity, meat quality, intestinal morphology and barrier function, cecum microbiota, and intestinal metabolome of rabbit. These attributes IOFP’s position as promising feed additives and as an potential alternatives to antibiotics, owing absence of antibiotics treated animals in the present study.

Dietary supplementation with IOFP had significantly improved the growth performance of chickens and ducks [11, 13]. However, its influence on rabbits remains unclear. In this study, administering 0.8% (w/w) IOFP along with the basal diet resulted in a significant increase in BW during the early period and improved feed efficiency throughout the trial period. Moreover, a 1.15-fold increase was observed in survival rate. A healthy gut functions is the foundation for maintaining and enhancing productivity. Dietary supplementation of *Epimedium* extract improved the growth performance of broilers by enhancing intestinal function [19]. Incorporating *Macleaya cordata* extract into the diet alleviates necrotic enteritis and enhances gut health in broilers, thereby promoting intestinal function and productivity [20]. Therefore, we assessed the effects of IOFP on intestinal morphology and function. First, we observed a significant alteration in intestinal morphology induced by IOFP, characterized by a reduction in crypt depth and an increase in the V/C value, which were closely correlated with intestinal development, maturation, digestion, and absorption. These results are consistent with those of previous studies [13, 16]. Second, IOFP significantly influenced intestinal barrier gene. The intestinal mucosa serves as the frontline defense against various invading pathogens, necessitating robust barrier function [6]. The mucosal mechanical barrier constitutes a crucial part of the mucosal barrier. Occludin and Claudin interact with ZO proteins that are linked to the actin cytoskeleton to establish an intestinal mechanical barrier that plays a pivotal role in paracellular permeability [21, 22]. Studies have confirmed that when the expression levels of barrier genes decrease, the permeability of intestinal epithelial cells increases, resulting in intestinal damage and susceptibility to pathogenic microbes [23]. In this study, the mRNA expression levels of *Claudin 1*, *Occludin*, *ZO1,* and *ZO2* genes were significantly upregulated, highlighting an enhancement in the integrity and functionality of the intestinal barrier. Therefore, the improvements in the F/G ratio and survival rate among rabbits in the IOFP-supplemented group were attributed to the enhanced development and functionality of the intestines.

The intestine serves not only as the primary digestive organ but also as the largest immune organ in the body. Therefore, enhancing immune function is crucial for improving productivity. Recent studies have shown that orally administered IOFP significantly improves the immune system in rabbits [9]. To further confirm the effect of IOFP on intestinal immune function, the intestinal sIgA content was assessed. sIgA-secreted by plasma cells in the lamina propria-is the most abundant and representative immunoglobulin on the mucosal surface. It serves to prevent microbial antigens from attaching to and entering the intestinal epithelium [24]. The results showed a significant increase in mucosal sIgA content in IOFP-fed rabbits compared to that of the control group, suggesting enhanced mucosal immune function. This improvement is particularly crucial during the transition from breastfeeding to solid-state feeding when the digestive system is not fully developed, contributing to high mortality rates among weaning.

One significant effect of *Inonotus obliquus* is antioxidant. It achieves this by either stimulating the activity of various antioxidant enzymes, such as SOD and CAT, or by minimizing the production of reactive oxygen species (ROS) [25–28]. Supplementing avian basal diets with IOFP significantly enhances the antioxidant activity of organisms [11, 13]. When IOFP was added to rabbit feed at the same concentration, a significant increase was observed in the activity/content of T-AOC, SOD, and NO in the serum and muscle. These findings suggest that *Inonotus obliquus* influences the antioxidant capacity of various animal species as antioxidant. Reports suggest that antioxidants added to animal feed can significantly influence carcass traits [29, 30], which direct correlates closely with economic benefits [3]. Thus, enhancing the carcass traits of meat rabbits is a vigorous action to incentivize workers and promote industrial growth. In this study, we examined the effect of IOFP on rabbit carcass traits. The slaughter ratio, influenced by factors such as breed, age, BW, nutrient composition, and feeding method of rabbits, showed a positive correlation with slaughter performance. The results showed that incorporating IOFP significantly improves slaughter performance metrics, including final BW, slaughter ratio, and commercial value. This suggests that IOFP supplementation increased conversion efficiency, and heightened antioxidant capacity.

As the nutritional benefits of rabbit meat gain awareness, it is increasingly becoming a part of the public diet. Ensuring superiority over other products and getting consumer favor hinges largely on meat quality, which directly influences consumption performance and potential nutritional value [31]. Antioxidants also play a role in influencing meat quality. One side, antioxidants reduce the buildup of oxidative by-products, thereby enhancing the pH value and properties of meat [32]. On the other side, a higher concentration of oxidative fibers leads to reduced glycolysis, which contributes to increased pH, redness, and water-holding capacity [5, 7]. In our study, we employed conventional meat quality indicators to assess the effects of IOFP. Color stands out as one of the most important attributes of fresh meat, playing a significant role in consumer purchasing decisions. Antioxidants play a role in regulating the redox state of myoglobin, thereby enhancing oxymyoglobin (OMb) content and rendering meat cherry-red color [3, 33], playing a significant role in consumer purchasing decisions. IOFP led to a significant increase in the redness value of the LTL muscle, resulting in a significant improvement in color. Water-holding capacity, crucial for meat quality, is determined by factors such as water and drop loss, which exhibit a negative correlation with water-holding capacity. When included as an antioxidant in the diet, IOFP significantly reduced drop and water loss, indicating promising improvement in meat quality. Tenderness was often assessed through shear force measurements. Lower shear force values show greater tenderness in meat [34]. In this study, IOFP significantly decreased shear force, ensuring the tenderness of fresh meat. Overall, dietary supplementation of IOFP in the basal diet significantly improved meat quality and nutritional value, by enhancing color, water-holding capacity, and tenderness, consequently enhancing its commercial value.

Due to the enhanced intestinal function and antioxidant activity facilitated by dietary IOFP, significant improvements in productivity, survival rates, and meat quality have been observed in rabbits. Intestinal health and function interact closely with the microbiome. Healthy gut microbiota is essential for maintaining gut homeostasis, facilitating nutrient digestion and absorption, and defending against invasion by pathogenic microorganisms [20, 35]. Moreover, there exists a significant correlation between the antioxidant capacity of organisms and their intestinal microbiome [36]. In current breeding and feeding practices, the supplement tends to exert a stronger influence on gut microbiota than breed, age, and sex; therefore, gut microbiota tends to adapt to changes in dietary ingredients, reflecting its dynamic nature [37]. The analysis of gut microbiota showed that dietary IOFP enhanced diversity and altered the composition of microbes, resulting in the regulation of many bacterial abundances. The IOFP group exhibited a significant decrease in the relative abundance of bacteria associated with intestinal disease, hyperglycemia/glucose metabolism disorder, and obesity than in the control group. Conversely, an increase was observed in bacteria, known for producing short-chain fatty acids (SCFA), immunopotentiating effects, antioxidation, hypoglycemic action, and degrading organic compounds. *Helicobacter*, *Parabacteroides*, *Ruminococcus_torques_group*, *Romboutsia*, *Colidextribacter*, and *Bilophila* are closely linked to enteritis. *Helicobacter* is specifically recognized as a primary pathogenic bacteria associated with colonists, significantly contributing to inflammatory bowel diseases (IBD) [38]. The abundance of *Parabacteroides* tends to rise in mice with dextran-sulfate-sodium (DSS) induced colitis. This increase is linked to a reduction in goblet cells and mucin 2 within the colon mucous layer, and the enhancement of intestinal permeability [23]. Enterotoxins represent another significant factor contributing to intestinal diseases. Hydrogen sulfide (H_2_S) can be generated through sulfates released by *Desulfovibrio*. This is toxic to intestinal epithelial cells and is implicated in the onset of gastrointestinal diseases [39]. Besides their influence on intestinal health, *Bilophila* and *Ruminococcus_torques_group* are associated with the regulation of host blood sugar levels. *Bilophila* exacerbates intestinal barrier dysfunction and abnormal bile acid metabolism induced by a high-fat diet in mice, resulting in more serious glucose metabolic dysfunction [40]. IOFP-treated animals exhibited a reduction in the relative abundance of pathogenic bacteria when compared to the control group. In addition, the levels of *Alistipes* and *Oscillibacter*, which are associated with obesity, also decreased [20, 41]. IOFP not only significantly decreased the relative abundance of pathogenic bacteria, but increased abundance of beneficial bacteria. In this study, IOFP significantly increased the abundance of *Porphyromonas*, a bacterium not detected in untreated rabbits. *Porphyromonas* is recognized as a producer of butyrate and acetic acid [42]. Besides, the abundance of *Sphingomonas* increased, which can convert pentosesan to acids and perform various biological functions [43]. Reports indicate that *Inonotus obliquus* is rich in flavonoids, demonstrating antioxidative properties, hypoglycemic action and immunopotentiating effect [44–46]. After 35 d of dietary IOFP supplementation, *Haliangium* and *MND1*, were not observed in untreated animals, whereas were found in the guts of treated rabbits. Studies have established a positive correlation between the two bacteria with flavonoids [47], suggesting a potential association with the enhancement of antioxidation, hypoglycemic action, and immunopotentiating effects observed in the IOFP group. However, further research is necessary to validate these findings. As herbivores, rabbits consume substantial amounts of indigestible substances daily, such as fiber and lignin. Improving the decomposition of these substances leads to a significant enhancement in feed efficiency. In the IOFP group, a significant increase in the relative abundances of *Comamonas* and *Sphingomonas* was observed than that in the control group. These two bacteria have been shown to degrade various organic materials, especially aromatic compounds [43, 48], thereby improving feed utilization and conversion. In addition, an increase in the relative abundance of *Phyllobacterium* was observed in the gut of IOFP-treated rabbits. It possesses the capability to decompose toxicants in hosts [49]. The synergy between *Comamonas*, *Sphingomonas,* and *Phyllobacterium* significantly enhanced the growth and health of rabbits in the IOFP group.

Gut microbiota influence host physiology by producing metabolites that regulate downstream signaling pathways and affect various physiological activities after absorption in the intestine [50]. Therefore, microbial metabolites serve as crucial intermediates linking microbial flora to their host. Changes in metabolites align with alterations in the composition or relative abundance of the gut microbiota [19]. In the present study, the metabolome was investigated to reveal the correlation between gut microbiota in the cecum and host physiology. Uric Acid is specifically known for its antioxidative properties in eliminating ROS [51]. Creatine, recognized as a nutrient and energy source, exhibits various biological functions such as improving energy metabolism, promoting muscle cell development, and antioxidant activity [52, 53]. Benzyl 6-O-beta-D-glucopyranosyl-beta-D-glucopyranoside exhibits physiological effects, including anticancer, hypolipemic, and hypoglycemic actions [54, 55]. All of metabolites were significantly increased. Moreover, some metabolites were significantly reduced, such as Stearoyl Ethanolamide, known to suppress appetite and production of NO synthetase [56], and Stearamide, which exhibits cytotoxic and tumorigenic [57]. All metabolites exhibiting significant differences between the groups primarily contribute to the enhancement of antioxidant activity, development, anti-inflammation, anti-depression, and lipid metabolism. They reduce fatty deposition, loss of appetite, and cytotoxicity. The results not only aligned with the improvement in growth performance, antioxidant activity, meat quality, and intestinal function, but also mirrored changes in the relative abundance of gut microbiota in rabbits. For instance, the numbers of *Alistipes* and *Desulfovibrio*, which are associated with depression and cytotoxicity to epithelial cells, respectively, was reduced [20, 47].

Pearson’s correlation analysis between significantly differentially expressed metabolites and microbes revealed that each metabolite exhibited a significant association with specific bacteria, with many being regulated by various bacteria. For example, Benzyl 6-O-beta-D-glucopyranosyl-beta-D-glucopyranoside, displayed significant positive associations with *Aeromicrobium*, *Bacillus*, *Bryobacter*, *Comamonas*, *Haliangium*, *MND1*, *Terrimonas*, and *UTCFX1*. Conversely, it exhibits significant negative correlations with *Colidextribacter* and *Lachnoclostridium*. The eight bacteria that notably positively correlated with the upregulated metabolites (Table 6) were exclusively detected in cecal digesta from rabbits fed with IOFP but not in untreated animals. These findings suggest that these microbes increased in population through IOFP supplementation in the basal diet. Therefore, IOFP improved the systemic function of rabbits and enhanced meat quality, likely through modulating the gut microbiota composition and abundance, leading to subsequent changes in availability of metabolites, while the causal relationships between these correlated metabolites and microbiota would be conducted in our future experiment.

## Conclusions

Supplementing 0.8% (w/w) IOFP in the basal diet introduced and enriched beneficial bacteria while reducing the abundance of pathogenic bacteria in weaning rabbits. This subsequently led to alterations in microbiota-derived metabolites. Alterations observed in microbiota and metabolites composition likely underline the improvement in barrier function, and mucosal immunity of the intestine in IOFP-treated animals. These changes contribute to enhanced growth performance and survival. In addition, the increased abundance of bacteria and metabolites associated with enhanced host antioxidant capicity significantly reinforces the antioxidant activity of rabbits, likely leading to improvements in carcass traits and meat quality. These findings suggest that IOFP could serve as an effective feed additive and a potential antibiotic alternative with more study is required for mammals.

## Methods

### Preparation and analysis of IOFP

*Inonotus obliquus* YU isolate was fermented by Qinhuangdao Gaotong Biotech Co., Ltd. (Changli, Hebei, China) in accordance with product regulations [12]. The product was stored at 4℃ until used.

The composition of IOFP was analyzed by UHPLC-MS/MS at Novogene Bioinformatics Technology Co., Ltd. (Beijing, China). Samples were injected into a C18 Hypesil Gold column (100 × 2.1 mm, 1.9 μm) (Thermo Fisher Scientific) using a 17 min linear gradient at a flow rate of 0.2 mL/min. The mobile phase for positive polarity mode were 0.1% formic acid (solvent A) and methanol (solvent B), while 5 mM ammonium acetate (pH 9.0) and methanol were used for negative polarity mode, as solvent A and B respectively. The solvent gradient was set as follows: 2% B, 1.5 min; 2–85% B, 3 min; 100% B, 10 min; 100–2% B, 10.1 min; 2% B, 12 min. Subsequently, Q Exactive HF-X mass spectrometer (Thermo Fisher Scientific) was used to analyze components in positive/negative polarity mode with spray voltage of 3.2 kV, capillary temperature of 320℃, sheath gas flow rate of 40 arb, aux gas flow rate of 10 arb, funnel RF level of 40, and aux gas heater temperature of 350 °C. All generated data were further processed using Compound Discover 3.1 (CD3.1) to perform peak alignment, peak picking, and quantitation for each metabolite. Then metabolites were matched with the mzCloud, mzVault and MassList database to obtain the accurate qualitative and relative quantitative results. Finally, KEGG, human metabolome database (HMDB) and LIPIDMaps database were used to identify metabolites by matching the molecular mass data.

### Animals and experimental design

Eighty healthy weaning Hyla rabbits (aged 35 d) with similar BWs were randomly divided into two groups [2]. Each group comprised 10 replicates, with four rabbits per replicate, ensuring equal distribution of males and females (each accounting for 50%). The control group was fed a basal diet. In contrast, the treatment group was orally administered IOFP at a dose of 0.8% (w/w) of the diet (concentration was determined as described in a previous study [9]), referred to as the IOFP group. The experiment spanned 35 d, during which the rabbits were subjected to routine feeding and management. Each rabbit was housed individually in cages and provided with food ad libitum. The basal diets provided in this study were formulated based on the recommended nutrient requirements for fattening rabbits [58] with some optimization. Table 7 presents the ingredients and calculated nutrient compositions.

**Table 7:** Ingredients and nutrient levels of diets (%, dry weight)

Item	Grower phase (d 35–70)
Ingredients	
Corn	5.00
Soybean meal	8.00
Barley	6.00
Wheat bran	15.00
Corn germ meal	16.00
Corn husk	17.00
Alfalfa meal	15.00
Soybean straw powder	7.00
Rice hull power	8.00
CaHPO_4_	1.50
NaCl	0.50
Premix^1^	1.00
Total	100.00
Nutrient levels	
DE/(MJ/kg)^2^	10.23
DM	89.82
CP	16.12
CF	17.38
NDF	38.74
ADF	23.08
ADL	6.29
EE	2.80
Ash	9.03
Ca	0.95
TP	0.45
Lys	0.60
Met + Cys	0.65

^1^Premix provided the following per kg of the diet: VA 10000 IU, VD_3_ 1500 IU, VE 50 mg, VK_3_ 3 mg, thiamine 5 mg, riboflavin 10 mg, pantothenic acid 20 mg, nicotinic acid 50 mg, Fe 100 mg, Zn 30 mg, Cu 20 mg, Mn 30 mg, Se 0.05 mg, choline 400 mg, NaCl 5 g, Lys 1 g, Met 1 g, the rest was miscellaneous meal carrier complement

^2^DE was calculated according to “Nutritional requirements of meat rabbits: NY/T 4049-2021” (Ministry of Agriculture and Rural Affairs of the People’s Republic of China, 2022), while the others were measured values

### Data record and sample collection

The initial BW of the rabbits in the IOFP and control groups was recorded at 35 d of age. Weekly measurements of BW and feed consumption were taken following 12 h of fasting. The ADG, ADFI, and F/G were calculated for each group. Both feed intake and F/G ratio were adjusted for mortality. The calculation formula referred to the method described in a previous study [11]. Mortality was determined based on daily observations of clinical manifestations and deaths among the rabbits. Blood samples were collected from both groups at 49 and 70 d of age, left to stand at room temperature for 4 h, and then centrifuged at 1,200 g for 15 min. The serum samples obtained were stored at − 20 °C until use. Following slaughter at 70 d of age, the carcass traits of both groups were assessed. The LTL muscles, located between the 1 st and 7th lumbar vertebra, were collected from both sides of each animal to assess their physical properties and muscle antioxidant activity. Partial intestines were fixed with 4% formaldehyde, while the remaining cecal specimens were stored at − 80 °C for the determination of sIgA levels, related genes expression levels, gut microbiota, and metabolites.

### Carcass traits and meat quality

At 70 d of age, after 12 h fast, rabbits from both groups were weighted and stunned using electric shock. After bloodletting, the commercial carcass weight, semi-clean, and full-clean carcass weight were determined post-treatment, as previously described [59]. Subsequently, the commercial slaughter ratio, semi-clean and full-clean slaughter ratio, was calculated by dividing their weights by their live weight before slaughter. Muscle color parameters, including brightness (L*), redness (a*), and yellowness (b*), were assessed within 30 min of slaughter using a tristimulus colorimeter (NR20XE, 3NH, Shenzhen, China) in the output mode. The calculated mean values were considered finals. Within 45–60 min of slaughter, a partial LTL sample was sliced into thin pieces measuring 1 cm. These slices were initially weighed (w1) and then reweighed (w2) after being subjected to 35 kg of pressure for 5 min. The water loss (%) was calculated using the formula: 100% × (w1-w2)/w1. Another section of the LTL muscle was cut into a square measuring 50 × 30 × 10 mm (length × width × thickness) and weighed (w3). It was then placed in a drop loss tube and stored in a refrigerator at 4 °C for 24 h. Finally, the final weight (w4) was measured to determine drop loss using the following equation: drop loss (%) = 100% × (w3-w4)/w3. Subsequently, the cooking loss of the LTL muscle specimens was assessed. The 30 g sample was cooked in a water bath at 90℃ for 45 min, and its weight was measured (w5) after cooling to room temperature. Cooking loss ratio (%) was calculated using the formula: 100% × (30 g-w5)/30 g. Most of the LTL muscle was refrigerated at 4℃. After 24 h, the pH value of the fifth rib of the LTL muscle was measured at three different locations using a portable pH meter (PHBJ-260, INESA, Shanghai, China), with the average value recorded as the pH_24h_. For another 24 h, the shear force (kgf) was tested using a muscle tenderness meter (C-LM, Tenovo, Harbin, China) as described previously [5].

### Antioxidant index of serum and muscle

T-AOC, SOD activity, and NO content were assessed in peripheral sera (49 and 70 d of age) and muscle samples (70 d of age) from IOFP-treated and untreated rabbits. This analysis was conducted using a commercial kit (ELISA method) manufactured by Nanjing Jiancheng Bioengineering Institute (Nanjing, China), following the manufacturer’s instructions.

### Intestinal morphology and sIgA concentration

Intestinal tissues, approximately 3 cm in length, were collected from rabbits in the treated and untreated groups. These tissues were washed with PBS and fixed with 4% paraformaldehyde. Tissue sections were prepared as previously described [16]. Five intact intestinal villus heights, crypt depths, and wall thicknesses were selected and measured from each tissue slice. The ratio of villus height to crypt depth was calculated and presented as mean value ± standard error of means (SEM). Partial intestinal tissues were treated with RIPA buffer (high) (Solarbio, Beijing, China), and the protein concentration was analyzed using a BCA protein assay kit (CW0014S, Cowin Biotech, Jiangsu, China). The sIgA concentration in the intestinal tissue was determined using an ELISA kit (Nanjing Jiancheng Bioengineering Institute, Nanjing, China) following the instructions of the manufacturer. Absorbances were recorded using a microplate reader (ELx800, BioTek Beijing, China) at a wavelength of 450 nm. The results were normalized to the protein concentration in each sample.

### Gene expression of tight junctions

Total RNA was extracted from the intestinal tissue using TRIzol reagent (Invitrogen, Carlsbad, CA, USA) and quantified using a NanoDrop 2000 (Thermo Fisher Scientific, Wilmington, DE, USA). Approximately 1 mg of RNA was used for synthesizing first-strand cDNA through reverse transcription, employing (dT)18 primers (TaKaRa Biotechnology, Dalian, China). Specific primers reported by Niu et al. [60] were employed for detecting the mRNA expression of rabbit *Claudin 1*, *Occludin*, *ZO1,* and *ZO2* using quantitative real-time PCR (qPCR). The fluorescence quantification kit used was TB Green Premix Ex Taq II (Tli RNase H Plus) (TaKaRa Biotechnology, Dalian, China). The reaction was conducted in a final volume of 20 μL, including 10 μL 2 × SYBR Premix Ex Taq II, 0.5 μg cDNA template, and 0.5 pmoL of each primer. The PCR cycling conditions were as follows: 1 cycle 95 °C for 1 min; 40 cycles of denaturation 95 °C for 15 s, annealing 60 °C for 25 s, extension 72℃ for 15 s, and a dissociation curve analysis step. Each sample was analyzed in triplicate. The relative gene expression levels were calculated using the 2^−∆∆Ct^ method to determine FC, with GADPH serving as the internal reference. To validate the assay, the purified PCR products were cloned into the pMD18-T plasmid and sequenced to confirm amplification.

### Microbial sequencing

Cecal digesta from 10 animals (one rabbit per replicate) from each group were collected to extract genomic DNA using the QIAamp DNA Stool Mini Kit (Qiagen, Valencia, CA, USA) according to the instructions of the manufacturer. The DNA concentration was measured using a NanoDrop 2000 (Thermo Fisher, Wilmington, DE, USA) and diluted to 1 ng/μL with double distilled water. The V3-V4 region of the 16S rRNA was amplified using barcoded primer pairs and Phusion High-Fidelity PCR Master Mix with GC buffer (NEB, Ipswich, MA, USA) to ensure efficiency and accuracy. The amplicons were mixed in equal proportions based on concentration and subsequently purified. Amplicon libraries were constructed, and sequence determination was conducted on the NovaSeq6000 platform by Novogene Bioinformatics Technology Co., Ltd. (Beijing, China).

The raw sequences underwent preprocessing and screening before analysis. FLASH (V1.2.7) software was used to merge paired-end reads to generate raw reads. Subsequently, the reads were truncated and filtered using Qiime (V1.9.1) based on specific criteria, and any chimeric sequences were removed. Finally, the effective tags were analyzed using Uparse (V7.0.1001) and clustered into operational taxonomic units (OTUs) at a similarity level of 97%. Subsequently, all representative reads were annotated using the Mothur and SSU rRNA databases in SILVA (version 138) employing an RDP classifier (with a 0.8–1 confidence threshold). Based on OTU clustering, alpha indices were used to characterize the diversity of cecum microbial species within each group. PCoA was employed to compare bacterial community structures across all samples. Moreover, the significance of the differentiation in microbial structures among the groups was statistically tested using similarity analysis. LDA was applied in conjunction with LEfSe to distinguish the bacteria between all treatments, with the LDA score set at four.

### Gut metabonomics

The cecal content, weighing 100 mg from each rabbit collected in the previous step, was mixed with 500 μL of 80% (v/v) methanol. The mixture was stored on ice for 5 min and then centrifuged at 10,000 × g for 20 min at 4 °C. The resulting supernatant was collected, and water was added to adjust the methanol content to 53%. After centrifugation at 15,000 g for 20 min at 4 °C, the supernatant was quantitatively measured using LC–MS/MS (Novogene Beijing, China). All data were introduced into the CD3.1 software to visualize metabolites and their relative abundance. They were further annotated using the KEGG, HMDB, and LIPIDMaps. Subsequently, data underwent additional processing by metaX before PCA, PLS-DA, and other analyses. Differential metabolites were identified using the t-test and VIP and further analyzed to gain additional biological information.

#### Statistical analysis

The growth performance, antioxidant capacity, meat quality, intestinal sIgA content, and mRNA expression levels of tight junction genes were analyzed using Student’s t-test with GraphPad Prism 8.0 (GraphPad Software, San Diego, CA, USA). Data were presented as mean and SEM. Mortality was assessed using Pearson’s Chi-Square test. A *P* value < 0.05 was considered statistically significant.

## Supplementary Information

Supplementary file 1.

## Data Availability

The 16S rRNA gene sequencing and metabolites data are available from the NGDC Genome Sequence Archive (CRA017936 (https://ngdc.cncb.ac.cn/gsa/s/rK0075Eu)) and Miscellaneous Data Archive (OMIX006970 (https://share.cncb.ac.cn/a119b1a2d3eb/)).

## Abbreviations

ADFI: Average daily feed intake
ADG: Average daily gain
BW: Body weights
DSS: Dextran-sulfate-sodium
FC: Fold change
F/G: Feed to gain ratio
HDL: High-density lipoprotein
IBD: Inflammatory bowel diseases
IOFP: *Inonotus obliquus* fermentation product
LDA: Linear discriminant analysis
LEfSe: Linear discriminant analysis effect size
LTL: Longissimus thoracis et lumborum
NE: Necrotic enteritis
NO: Nitric oxide
OTUs: Operational taxonomic units
PCA: Principal component analysis
PCoA: Principal coordinate analysis
PLS-DA: Partial least squares discriminant analysis
qPCR: Quantitative real-time PCR
ROS: Reactive oxygen species
SCFA: Short-chain fatty acids
SEM: Standard error of means
sIgA: Secrete IgA
SOD: Superoxide dismutase
T-AOC: Total antioxidant capacity
V/C: Villus height to crypt depth ratio
