# Supplementary material for: Inonotus obliquus fermentation product improves growth performance and meat quality probably through intestine and antioxidant capacity enhanced by gut microbes and metabolites regulation in rabbits
Source: Anim Microbiome. 2025 Jun 9;7:61. doi: 10.1186/s42523-025-00427-7 (PMC12147337; doi:10.1186/s42523-025-00427-7)
Supplement: Supplementary file 1 [file 42523_2025_427_MOESM1_ESM.doc]

**Supplementary Table S1** Compounds in IOFP

| ***NO.*** | ***Compound*** | ***RT (min)*** | ***Molecular formula*** | ***Molecular Weight*** | ***NO.*** | ***Compound*** | ***RT (min)*** | ***Molecular formula*** | ***Molecular Weight*** |
| --- | --- | --- | --- | --- | --- | --- | --- | --- | --- |
| 1 | Menaquinone | 11.989 | C_31_H_40_O_2_ | 444.3026 | 105 | 2-Hydroxycinnamic acid | 2.303 | C_9_H_8_O_3_ | 164.0476 |
| 2 | Cyclohexaneacetic acid | 11.975 | C_8_H_14_O_2_ | 142.0987 | 106 | N-Acetyl-1-aspartylglutamic acid | 2.252 | C_11_H_16_N_2_O_8_ | 304.0903 |
| 3 | 3-Methylhistidine | 11.967 | C_7_H_11_N_3_O_2_ | 169.0858 | 107 | N-Acetyl-L-methionine | 2.23 | C_7_H_13_NO_3_S | 191.0622 |
| 4 | LPK | 11.878 | C_17_H_32_N_4_O_4_ | 356.2436 | 108 | Nicotinamide | 2.224 | C_6_H_6_N_2_O | 122.0484 |
| 5 | L-Lysine | 11.870 | C_6_H_14_N_2_O_2_ | 146.1061 | 109 | Levodopa | 2.208 | C_9_H_11_NO_4_ | 197.0694 |
| 6 | (±)12(13)-DiHOME | 7.294 | C_18_H_34_O_4_ | 314.2455 | 110 | 3-Indoleacrylic acid | 2.208 | C_11_H_9_NO_2_ | 187.0633 |
| 7 | Hydrocortisone | 6.706 | C_21_H_30_O_5_ | 362.2097 | 111 | N2-Methylguanosine | 2.158 | C_11_H_15_N_5_O_5_ | 297.1078 |
| 8 | Oxymatrine | 6.684 | C_15_H_24_N_2_O_2_ | 264.1842 | 112 | N7-Methylguanosine | 2.154 | C_11_H_17_N_5_O_5_ | 299.1219 |
| 9 | Sedanolide | 6.536 | C_12_H_18_O_2_ | 194.1312 | 113 | N-Acetyl-DL-glutamic acid | 2.122 | C_7_H_11_NO_5_ | 189.0633 |
| 10 | Hexadecanedioic acid | 6.517 | C_16_H_30_O_4_ | 286.214 | 114 | Uridine monophosphate (UMP) | 2.117 | C_9_H_13_N_2_O_9_P | 324.0354 |
| 11 | Minocycline | 6.447 | C_23_H_27_N_3_O_7_ | 457.1863 | 115 | cis-Aconitic acid | 2.111 | C_6_H_6_O_6_ | 174.0160 |
| 12 | Bestatin | 6.43 | C_16_H_24_N_2_O_4_ | 308.173 | 116 | J147 | 2.109 | C_18_H_17_F_3_N_2_O_2_ | 350.1233 |
| 13 | Traumatic acid | 6.395 | C_12_H_20_O_4_ | 228.1368 | 117 | Epinephrine bitartrate | 2.089 | C_13_H_19_NO_9_ | 333.1072 |
| 14 | Undecanedioic acid | 6.326 | C_11_H_20_O_4_ | 216.1356 | 118 | 10-Undecenoic acid | 2.084 | C_11_H_20_O_2_ | 184.1466 |
| 15 | Benzoic acid | 6.286 | C_7_H_6_O_2_ | 122.0363 | 119 | Gluconolactone | 2.066 | C_6_H_10_O_6_ | 178.0484 |
| 16 | Cuminaldehyde | 6.145 | C_10_H_12_O | 148.0892 | 120 | Hypoxanthine | 2.064 | C_5_H_4_N_4_O | 136.0389 |
| 17 | Sebacic acid | 6.114 | C_10_H_18_O_4_ | 202.1201 | 121 | DL-Panthenol | 2.002 | C_9_H_19_NO_4_ | 205.1318 |
| 18 | 6-Aminonicotinamide | 6.058 | C_6_H_7_N_3_O | 137.0589 | 122 | Lactobionic acid | 2.001 | C_12_H_22_O_12_ | 358.1112 |
| 19 | Kynurenic acid | 6.03 | C_10_H_7_NO_3_ | 189.0431 | 123 | 5-Methylcytosine | 1.998 | C_5_H_7_N_3_O | 125.0593 |
| 20 | Tetramethylpyrazine | 6.015 | C_8_H_12_N_2_ | 136.1003 | 124 | L-Pyroglutamic acid | 1.992 | C_5_H_7_NO_3_ | 129.0429 |
| 21 | Propionyl-L-carnitine | 6 | C_10_H_19_NO_4_ | 217.1308 | 125 | Methionine | 1.966 | C_5_H_11_NO_2_S | 149.0514 |
| 22 | Oleoyl-L-alpha-lysophosphatidic acid | 5.93 | C_21_H_41_O_7_P | 436.2583 | 126 | Deoxyinosine | 1.963 | C_10_H_12_N_4_O_4_ | 252.0864 |
| 23 | Hippuric acid | 5.925 | C_9_H_9_NO_3_ | 179.0577 | 127 | 2-Deoxyuridine | 1.907 | C_9_H_12_N_2_O_5_ | 228.075 |
| 24 | Azelaic acid | 5.919 | C_9_H_16_O_4_ | 188.1044 | 128 | 5-Aminovaleric acid | 1.881 | C_5_H_11_NO_2_ | 117.0785 |
| 25 | L-Adrenaline | 5.903 | C_9_H_13_NO_3_ | 183.0893 | 129 | Uric acid | 1.874 | C_5_H_4_N_4_O_3_ | 168.02786 |
| 26 | DL-o-Tyrosine | 5.898 | C_9_H_11_NO_3_ | 181.0734 | 130 | Pseudouridine | 1.863 | C_9_H_12_N_2_O_6_ | 244.0691 |
| 27 | Diflorasone | 5.841 | C_22_H_28_F_2_O_5_ | 410.1909 | 131 | Dihydrothymine | 1.852 | C_5_H_8_N_2_O_2_ | 128.0589 |
| 28 | D(+)-Phenyllactic acid | 5.814 | C_9_H_10_O_3_ | 166.0625 | 132 | 5-Methyl-dl-tryptophan | 1.84 | C_12_H_14_N_2_O_2_ | 218.1047 |
| 29 | 3-Methoxytyramine | 5.797 | C_9_H_13_NO_2_ | 167.095 | 133 | 2-Furoic acid | 1.837 | C_5_H_4_O_3_ | 112.016 |
| 30 | Glu-Val-Phe | 5.78 | C_19_H_27_N_3_O_6_ | 393.1908 | 134 | Uracil | 1.833 | C_4_H_4_N_2_O_2_ | 112.0277 |
| 31 | Syringic acid | 5.772 | C_9_H_10_O_5_ | 198.0525 | 135 | D-α-Hydroxyglutaric acid | 1.830 | C_5_H_8_O_5_ | 148.0368 |
| 32 | Phenylpyruvic acid | 5.734 | C_9_H_8_O_3_ | 164.0471 | 136 | Pyridoxine | 1.805 | C_8_H_11_NO_3_ | 169.0742 |
| 33 | 9-Aminoacridine | 5.702 | C_13_H_10_N_2_ | 194.0851 | 137 | Citric acid | 1.775 | C_6_H_8_O_7_ | 192.0265 |
| 34 | Kinetin 9-riboside | 5.681 | C_15_H_17_N_5_O_5_ | 347.1217 | 138 | N-Acetylaspartic acid | 1.738 | C_6_H_9_NO_5_ | 175.0477 |
| 35 | CYM-5442 | 5.669 | C_23_H_27_N_3_O_4_ | 409.2009 | 139 | 4-Hydroxyisoleucine | 1.722 | C_6_H_13_NO_3_ | 147.0898 |
| 36 | Caffeic acid | 5.632 | C_9_H_8_O_4_ | 180.0420 | 140 | cGMP | 1.666 | C_10_H_12_N_5_O_7_P | 345.0475 |
| 37 | 5-Methyltetrahydrofolic acid | 5.621 | C_20_H_25_N_7_O_6_ | 459.1850 | 141 | Guanosine monophosphate | 1.665 | C_10_H_14_N_5_O_8_P | 363.0581 |
| 38 | 4-Methylphenol | 5.618 | C_7_H_8_O | 108.0573 | 142 | 2-Oxoglutaric acid | 1.665 | C_5_H_6_O_5_ | 146.0211 |
| 39 | L-Tryptophan | 5.586 | C_11_H_12_N_2_O_2_ | 204.0894 | 143 | 4-Guanidinobutyric acid | 1.643 | C_5_H_11_N_3_O_2_ | 145.0854 |
| 40 | 3-[(4-hydroxyphenyl)methyl]-octahydropyrrolo  [1,2-a]pyrazine-1,4-dionemaculosin | 5.579 | C_14_H_16_N_2_O_3_ | 260.1167 | 144 | Valine | 1.635 | C_5_H_11_NO_2_ | 117.0793 |
| 41 | Bz-RS-ISer(3-Ph)-Ome | 5.561 | C_17_H_17_NO_4_ | 299.1163 | 145 | Pyroglutamic acid | 1.663 | C_5_H_7_NO_3_ | 129.043 |
| 42 | Virginiamycin | 5.495 | C_28_H_35_N_3_O_7_ | 525.2477 | 146 | S-Methyl-L-cysteine | 1.633 | C_4_H_9_NO_2_S | 135.0357 |
| 43 | Terephthalic acid | 5.492 | C_8_H_6_O_4_ | 166.0261 | 147 | Cytosine | 1.617 | C_4_H_5_N_3_O | 111.0437 |
| 44 | 3-(propan-2-yl)-octahydropyrrolo  [1,2-a]pyrazine-1,4-dione | 5.458 | C_10_H_16_N_2_O_2_ | 196.1217 | 148 | Cytidine | 1.616 | C_9_H_13_N_3_O_5_ | 243.0859 |
| 45 | N-Acetylmethionine | 5.369 | C_7_H_13_NO_3_S | 191.0622 | 149 | Malonic acid | 1.61 | C_3_H_4_O_4_ | 104.0107 |
| 46 | Vitamin B2 | 5.297 | C_17_H_20_N_4_O_6_ | 376.1391 | 150 | trans-Aconitic acid | 1.607 | C_6_H_6_O_6_ | 174.0160 |
| 47 | 5'-S-Methyl-5'-thioadenosine | 5.271 | C_11_H_15_N_5_O_3_S | 297.0903 | 151 | UMP | 1.601 | C_9_H_13_N_2_O_9_P | 324.0358 |
| 48 | Homovanillic acid | 5.264 | C_9_H_10_O_4_ | 182.0577 | 152 | Citicoline | 1.581 | C_14_H_26_N_4_O_11_P_2_ | 488.1056 |
| 49 | DL-Tryptophan | 5.240 | C_11_H_12_N_2_O_2_ | 204.0897 | 153 | L-Threonine | 1.579 | C_4_H_9_NO_3_ | 119.0586 |
| 50 | Leucylproline | 5.181 | C_11_H_20_N_2_O_3_ | 228.1481 | 154 | Oxacepro | 1.574 | C_7_H_11_NO_4_ | 173.0692 |
| 51 | Bilirubin | 5.17 | C_33_H_36_N_4_O_6_ | 584.2621 | 155 | D-Threose | 1.573 | C_4_H_8_O_4_ | 120.0425 |
| 52 | Chelidamic acid hydrate | 5.133 | C_7_H_5_NO_5_ | 183.0166 | 156 | Dulcitol | 1.564 | C_6_H_14_O_6_ | 182.0785 |
| 53 | Glycyl-L-leucine | 5.058 | C_8_H_16_N_2_O_3_ | 188.1166 | 157 | Fumaric acid | 1.556 | C_4_H_4_O_4_ | 116.0105 |
| 54 | 3-Methylindole | 5.047 | C_9_H_9_N | 131.0739 | 158 | DL-Malic acid | 1.555 | C_4_H_6_O_5_ | 134.0211 |
| 55 | D-Phenylalanine | 5.044 | C_9_H_11_NO_2_ | 165.0787 | 159 | 4-Pyridoxic acid | 1.543 | C_8_H_9_NO_4_ | 183.0535 |
| 56 | Physostigmine | 5.038 | C_15_H_21_N_3_O_2_ | 275.1641 | 160 | N-Acetylornithine | 1.540 | C_7_H_14_N_2_O_3_ | 174.1008 |
| 57 | Royal jelly acid | 5.036 | C_10_H_18_O_3_ | 186.1248 | 161 | D-Proline | 1.539 | C_5_H_9_NO_2_ | 115.063 |
| 58 | Glycocholic acid hydrate | 4.999 | C_26_H_45_NO_7_ | 483.3184 | 162 | L-Cystathionine | 1.531 | C_7_H_14_N_2_O_4_S | 222.0671 |
| 59 | Pyridoxamine | 4.985 | C_8_H_12_N_2_O_2_ | 168.0901 | 163 | L-Dihydroorotic Acid | 1.51 | C_5_H_6_N_2_O_4_ | 158.0324 |
| 60 | Biotin | 4.951 | C_10_H_16_N_2_O_3_S | 244.0887 | 164 | N-Methylhydantoin | 1.502 | C_4_H_6_N_2_O_2_ | 114.0426 |
| 61 | 2'-O-Methyladenosine | 4.925 | C_11_H_15_N_5_O_4_ | 281.1133 | 165 | Ergothioneine | 1.501 | C_9_H_15_N_3_O_2_S | 229.0888 |
| 62 | 2'-O-Methyluridine | 4.837 | C_10_H_14_N_2_O_6_ | 258.0845 | 166 | JNJ-1661010 | 1.474 | C_19_H_19_N_5_OS | 365.1324 |
| 63 | Leucine-enkephalin | 4.836 | C_28_H_37_N_5_O_7_ | 555.2677 | 167 | Choline Glycerophosphate | 1.472 | C_8_H_20_NO_6_P | 257.1016 |
| 64 | Spectinomycin | 4.715 | C_14_H_24_N_2_O_7_ | 332.1586 | 168 | Proline | 1.471 | C_5_H_9_NO_2_ | 115.0636 |
| 65 | L-Kynurenine | 4.643 | C_10_H_12_N_2_O_3_ | 208.0856 | 169 | Acetylcholine | 1.464 | C_7_H_15_NO_2_ | 145.1106 |
| 66 | Xanthosine | 4.313 | C_10_H_12_N_4_O_6_ | 284.0755 | 170 | Noradrenaline | 1.464 | C_8_H_11_NO_3_ | 169.0734 |
| 67 | NVP-231 | 4.126 | C_25_H_25_N_3_O_2_S | 431.1652 | 171 | N-acetyl-D-glucosamine | 1.454 | C_8_H_15_NO_6_ | 221.0902 |
| 68 | L-Ascorbate | 4.101 | C_6_H_8_O_6_ | 176.0318 | 172 | N-Acetylneuraminic acid | 1.450 | C_11_H_19_NO_9_ | 309.1064 |
| 69 | 2'-Deoxyinosine | 4.075 | C_10_H_12_N_4_O_4_ | 252.0849 | 173 | Creatine | 1.444 | C_4_H_9_N_3_O_2_ | 131.0697 |
| 70 | cAMP | 4.063 | C_10_H_12_N_5_O_6_P | 329.0523 | 174 | D-Glucosamine 6-phosphate | 1.439 | C_6_H_14_NO_8_P | 259.0462 |
| 71 | 8-Hydroxyguanosine | 4.056 | C_10_H_13_N_5_O_6_ | 299.0861 | 175 | Choline bitartrate | 1.435 | C_9_H_19_NO_7_ | 253.1166 |
| 72 | Histamine | 3.775 | C_5_H_9_N_3_ | 111.0802 | 176 | Sucrose | 1.435 | C_12_H_22_O_11_ | 342.1157 |
| 73 | Serotonin | 3.754 | C_10_H_12_N_2_O | 176.0946 | 177 | Xylitol | 1.432 | C_5_H_12_O_5_ | 152.0687 |
| 74 | Nicotinic acid | 3.748 | C_6_H_5_NO_2_ | 123.0317 | 178 | DL-Lysine | 1.431 | C_6_H_14_N_2_O_2_ | 146.1057 |
| 75 | L-5-Hydroxytryptophan | 3.741 | C_11_H_12_N_2_O_3_ | 220.0852 | 179 | Caffeine | 1.431 | C_8_H_10_N_4_O_2_ | 194.0807 |
| 76 | Estropipate | 3.696 | C_22_H_32_N_2_O_5_S | 436.2051 | 180 | Muramic acid | 1.430 | C_9_H_17_NO_7_ | 251.1012 |
| 77 | Alanyltyrosine | 3.610 | C_12_H_16_N_2_O_4_ | 252.1117 | 181 | D-(-)-Glutamine | 1.428 | C_5_H_10_N_2_O_3_ | 146.0688 |
| 78 | O-Acetyl-L-carnitine | 3.555 | C_9_H_17_NO_4_ | 203.1152 | 182 | Trigonelline | 1.423 | C_7_H_7_NO_2_ | 137.0479 |
| 79 | 2,6-Dihydroxypurine | 3.516 | C_5_H_4_N_4_O_2_ | 152.0336 | 183 | 7-Methylxanthine | 1.423 | C_6_H_6_N_4_O_2_ | 166.0495 |
| 80 | Guanine | 3.511 | C_5_H_5_N_5_O | 151.0496 | 184 | Gluconic acid | 1.419 | C_6_H_12_O_7_ | 196.0579 |
| 81 | L-Fucose | 3.489 | C_6_H_12_O_5_ | 164.0692 | 185 | DL-Carnitine | 1.415 | C_7_H_15_NO_3_ | 161.1054 |
| 82 | Gly-Tyr | 3.487 | C_11_H_14_N_2_O_4_ | 238.0957 | 186 | L-Histidine | 1.415 | C_6_H_9_N_3_O_2_ | 155.0697 |
| 83 | Adenine | 3.410 | C_5_H_5_N_5_ | 135.0542 | 187 | Choline | 1.410 | C_5_H_13_NO | 103.1002 |
| 84 | Adenosine | 3.409 | C_10_H_13_N_5_O_4_ | 267.0970 | 188 | 4-Acetamidobutyric acid | 1.406 | C_6_H_11_NO_3_ | 145.0741 |
| 85 | Isoproterenol | 3.284 | C_11_H_17_NO_3_ | 211.1212 | 189 | L-Anserine | 1.405 | C_10_H_16_N_4_O_3_ | 240.1218 |
| 86 | Thymidine | 3.061 | C_10_H_14_N_2_O_5_ | 242.0899 | 190 | Threonine | 1.394 | C_4_H_9_NO_3_ | 119.0580 |
| 87 | 2'-Deoxyuridine | 3.046 | C_9_H_12_N_2_O_5_ | 228.0741 | 191 | Citrulline | 1.398 | C_6_H_13_N_3_O_3_ | 175.0954 |
| 88 | L-Norleucine | 3.013 | C_6_H_13_NO_2_ | 131.0948 | 192 | L-Ornithine | 1.397 | C_5_H_12_N_2_O_2_ | 132.0895 |
| 89 | Pilocarpine | 2.961 | C_11_H_16_N_2_O_2_ | 208.1215 | 193 | 3-Hydroxy-3-methylglutaric acid | 1.387 | C_6_H_10_O_5_ | 162.0525 |
| 90 | Pantethine | 2.827 | C_22_H_42_N_4_O_8_S_2_ | 554.2463 | 194 | L-Glutamic acid | 1.377 | C_5_H_9_NO_4_ | 147.0529 |
| 91 | Lactitol | 2.762 | C_12_H_24_O_11_ | 344.1326 | 195 | Glutaric Acid | 1.377 | C_5_H_8_O_4_ | 132.0418 |
| 92 | Mevalonic acid | 2.708 | C_6_H_12_O_4_ | 148.0732 | 196 | D-(-)-Mannitol | 1.372 | C_6_H_14_O_6_ | 182.0785 |
| 93 | Paracetamol | 2.593 | C_8_H_9_NO_2_ | 151.0637 | 197 | Allantoin | 1.319 | C_4_H_6_N_4_O_3_ | 158.0433 |
| 94 | 3'-O-Methylcytidine | 2.583 | C_10_H_15_N_3_O_5_ | 257.1017 | 198 | Hypotaurine | 1.318 | C_2_H_7_NO_2_S | 109.02 |
| 95 | Dehydrocholic acid | 2.548 | C_24_H_34_O_5_ | 402.2424 | 199 | Taurine | 1.307 | C_2_H_7_NO_3_S | 125.0144 |
| 96 | 5-Hydroxyindole | 2.505 | C_8_H_7_NO | 133.0531 | 200 | DL-Serine | 1.293 | C_3_H_7_NO_3_ | 105.0425 |
| 97 | Tyramine | 2.454 | C_8_H_11_NO | 137.0835 | 201 | L-Aspartic acid | 1.269 | C_4_H_7_NO_4_ | 133.0373 |
| 98 | Succinic acid | 2.440 | C_4_H_6_O_4_ | 118.0263 | 202 | Ornithine | 1.181 | C_5_H_12_N_2_O_2_ | 132.0902 |
| 99 | Coumarin | 2.314 | C_9_H_6_O_2_ | 146.0370 | 203 | D-glucuronic acid | 1.128 | C_6_H_10_O_7_ | 194.0433 |
| 100 | L-Tyrosine | 2.313 | C_9_H_11_NO_3_ | 181.0741 | 204 | Inosine | 1.127 | C_10_H_12_N_4_O_5_ | 268.08 |
| 101 | Acetophenone | 2.312 | C_8_H_8_O | 120.0579 | 205 | Deoxyguanosine | 1.123 | C_10_H_13_N_5_O_4_ | 267.0961 |
| 102 | 4-Hydroxybenzaldehyde | 2.308 | C_7_H_6_O_2_ | 122.0372 | 206 | Pyrogallol | 0.951 | C_6_H_6_O_3_ | 126.0320 |
| 103 | Uridine | 2.308 | C_9_H_12_N_2_O_6_ | 244.0691 | 207 | Quinolinic acid | 0.544 | C_7_H_5_NO_4_ | 167.0215 |
| 104 | 5-Methyluridine | 2.304 | C_10_H_14_N_2_O_6_ | 258.0847 | 208 | L-arginine | 0.529 | C_6_H_14_N_4_O_2_ | 174.112 |
